# Prematurity: An Overview and Public Health Impacts of Being Born Too Early

**DOI:** 10.1177/2333794X20987779

**Published:** 2021-01-11

**Authors:** Lulu M. Muhe, Assaye K. Nigussie, Amha Mekasha, Bogale Worku, Meseret Zelalem

**Affiliations:** 1 Addis Ababa University, Addis Ababa, Ethiopia; 2 Bill and Melinda Gates Foundation, Seattle, USA (retired)

National estimates of preterm birth (defined as childbirth at less than 37 completed weeks of gestation) for 194 WHO member countries have been published for the year 2014 showing nearly 15 million preterm births.^1^ Over 81% of preterm births occur in sub-Saharan Africa and South Asia.^1^ Top 10 countries contribute to 56% of the world’s preterm babies and Ethiopia belongs to this group of countries, contributing to 2.5% of the global burden.^1^ Evidence from both developed and developing countries that maintain good birth registries shows that the burden of preterm birth is increasing.^2,3^ Factors possibly contributing to, but not completely explaining this upward trend, include increasing rates of multiple births, greater use of assisted reproduction techniques, increases in the proportion of births among women over 34 years of age and changes in clinical practices, such as greater use of labour induction and elective Caesarean section.

Complications from preterm births are the leading direct cause of neonatal deaths accounting for 35% of all newborn deaths. Around 47% of under-five deaths that is, just over one third of the 2.5 million annual newborn deaths result from preterm-related causes.^4^ Mortality rates increase proportionally with decreasing gestational age or birth weight.^5^ In Ethiopia, of the estimated 91 700 neonatal deaths in 2010, a little more than one-third of them were believed to be due to preterm complications.

Historically, prematurity-related complications, referred to as “prematurity” have been described just like one entity among causes of under-five and neonatal mortality.^6^ However, preterm neonatal deaths could be related to either preterm-related complications that are specific to the fact that the neonate is born too soon or the deaths could be due to conditions such as congenital anomalies, asphyxia or sepsis that may also cause death in the term infant. Common preterm causes of death include: Respiratory Distress Syndrome (RDS), Necrotizing Enterocolitis (NEC), Intraventricular Haemorrhage (IVH) and metabolic complications (hypothermia, hypoglycaemia, hypocalcaemia). “The WHO ICD-10 requests that clinicians do not enter prematurity as the main disease or condition in the foetus or infant unless it was the only fetal or infant condition known to cause the death. There is a tendency in many settings to assign prematurity as a direct cause of death, when further evidence to suggest a more definitive cause of death has not been actively sought.”^7^

It was with the above background that Addis Ababa University in collaboration with Gondar University and Jimma University had taken the initiative to study the major causes of illness and death in preterm babies. The study was primarily designed to unpack the so-called preterm complications and come up with a new pie-chart that is showing the major underlying and contributing causes of death in preterm babies. The pie-chart is the first of its kind in Ethiopia and is pivotal to guide the national policy and the newborn care practice to further reduce neonatal mortality and morbidity in Ethiopia

The Study of Illness of Preterms (SIP) was therefore, a multi-center prospective observational study undertaken in 5 hospitals in Ethiopia over a period of nearly 2 years, between 2016 and 2018 to determine the major causes of preterm mortality in the first 28 days of life.^8^ Data were collected on the maternal/obstetric history, clinical maternal and neonatal conditions, laboratory and radiological investigations. In addition, for those who died, consent was requested for post-mortem examinations (both complete diagnostic autopsy [CDA] and minimally invasive tissue sampling [MITS]). An independent panel of experts composed of international and national newborn health experts determined the primary and contributory causes of preterm mortality based on available data and criteria that were developed a priori. Nearly 5000 preterm infants were enrolled in the study and by 28 days of postnatal age, 29% (1109) of those admitted to the NICU died. CDA was done in 441 (40%) and MITS was done in 126 (11%) of the NICU deaths.^8^

The SIP study found that RDS (45%), sepsis, pneumonia and meningitis (combined as neonatal infections, 30%) and asphyxia (14%) as the major causes of death in preterm infants. The highest mortality rate occurred among infants below 28 weeks of gestation (86%), followed by those between 28 and 31 weeks (54%), 32 and 34 weeks (18%) and in those 34 to 36 weeks (8%).^8^

As part of the investigations to support determination of cause of death, we did both CDA and MITS and compared them to each other. We showed that the minimally invasive tissue sampling (MITS) method can sample adequate core tissue for histopathological studies equivalent to the standard autopsy for most newborn conditions in preterm infants including for lung pathologies (respiratory distress syndrome, pulmonary hemorrhage) and hepatic lesions.^9^ The MITS method was deficient in obtaining sufficient core tissue of the intestine and brain.^9^ Among the 441 autopsies of preterm neonatal deaths, Hyaline Membrane Disease (HMD) was recorded in 81.6%, Pneumonia in 44.7%, pulmonary haemorrhage or diffuse alveolar haemorrhage in 39%, and meconium aspiration in 5.9%.^10^ HMD was conspicuous in the extremely and moderately preterm infants and that might have contributed to a higher death rate in these groups of babies.^10^

We studied maternal and neonatal factors that might have contributed to the high preterm mortality. Maternal factors such as low education status, presence of maternal infection, lack of ANC check-up were significantly associated to mortality of the preterm infant.^11^ Even though pregnancy induced hypertensive disorders such as pre-eclampsia and eclampsia were present in 20% of the pregnant women, it was not significantly associated with preterm mortality. Neonatal factors such as multiple gestations and male sex were associated with preterm mortality.^11,12^

We also monitored feeding patterns of these infants while staying in NICUs. Enteral feeding of the infants was started on the second and fourth day for 75.7% and 21.8% of the infants respectively.^13^ The proportion of infants on breast milk only, preterm formula, term formula and mixed feeding was 58%, 27.4%, 1.6%, and 34.1% respectively. Delay in enteral feeding was associated with increased risk of death, (OR = 1.92, 95% CI 1.33, 2.78) and (OR 5.06, 95% CI 3.23, 7.87, *P* = .000) for 1 to 3 and 4 to 6 days of delay in enteral feeding respectively after adjusting for possible confounders.^13^

Hypothermia was designated, by the panel’s prior definition, as a contributory cause since it was commonly observed together with almost all other primary causes of death. The SIP study showed that hypothermia is a common condition among preterms (69%) and it contributes to preterm mortality; the lower the body temperature, the higher was the mortality rate.^14^

Sepsis was one of the major causes of preterm deaths as determined by the panel. Among infants with the diagnosis of sepsis, Klebsiella species, Coagulase negative Staphylococcus and Staphylococcus *aureus* were the predominant pathogens isolated by blood culture.^15^ Ciprofloxacin was the most effective drug against the Gram-positive and Gram-negative bacteria, whereas resistance to the more commonly used antibiotics such as; ampicillin and gentamycin was very high, more than 80%.^15^ Other investigations such as complete blood count and bilirubin were done in most cases. Abnormal WBC and platelet counts were the most common to be associated with Early Onset Neonatal Sepsis (EONS), asphyxia and RDS.^16^ The prevalence of hyperbilirubinemia in preterm babies admitted to neonatal care units in Ethiopia was high. In addition to immature bilirubin metabolism commonly seen in preterm babies, the major risk factors associated with hyperbilirubinemia in preterm babies in this study were found to be ABO incompatibility, sepsis and Rh isoimmunisation.^17^

One of the reasons why the team has embarked upon doing this study was primarily because the rate of reduction of neonatal mortality has been minimal in Ethiopia and in many low resource settings. In the past 5 years, neonatal mortality refused to decline in Ethiopia. In fact, it has slightly moved to the higher side, from 29/1000 LB in 2016 mini-EDHS to 30/1000LB in 2019 EMDHS. Preterm mortality contributes to nearly one-third of the neonatal mortality. Therefore, for Ethiopia to further bend the neonatal mortality curve, there is a need to scale up the proven preventive and curative neonatal interventions to tackle the common causes of preterm mortality, such as respiratory distress syndrome, sepsis and asphyxia. Interventions to improve preterm birth outcomes such as antenatal corticosteroids to improve neonatal lung maturation, skin-to-skin and continuous KMC to tackle hypothermia, essential newborn care including resuscitation of asphyxiated babies, continuous positive airway pressure for RDS, and optimal infant feeding are recommended globally. However, the coverage of these interventions has often been very low and of poor quality. For example, this study has shown that corticosteroids were used for only 700 (31.2%) pregnant women out of 2243 whose gestational age was between 24 to 34 weeks.^18^

## Public Health Implications and a Call for Action

The findings of the SIP study showed that RDS, sepsis and asphyxia are the major causes of preterm mortality. The study has highlighted the challenges that exist in the WHO ICD-10 disease classification of COD of preterms and the need to strengthen capacity of health professionals on the diagnosis and classification of COD previously lumped together as “prematurity.” This study also highlights the importance of scaling up the known high impact interventions to prevent or treat the main causes of death in preterm babies. Based on the findings of this study, it may be prudent to have a policy dialogue with policy makers to make sure that the necessary policy and practice- change including review and revision of hospital clinical management protocols are happening. It will also be imperative to increase investments for newborn health to be able to introduce new and high impact interventions and to scale up the coverage of the proven interventions targeting the major causes of preterm mortality to meet the SDG target of reducing neonatal mortality rate to below 12 per 1000 LB. Further research is encouraged and called for to confirm the findings of this study and to develop effective and affordable intervention packages to prevent and treat the major causes of preterm death. The study team is therefore, recommending the following action points as a way forward:

1. To strengthen the supportive infrastructure and high impact low cost interventions including skin-to-skin thermal care (KMC), early and exclusive breastfeeding, parenteral nutrition, blended oxygen for preterms and expand/strengthen warm chain system.
2. To improve capacity of health professionals to provide adequate and advanced inpatient quality newborn care.
3. To strengthen and improve treatment of the common causes of preterm neonatal deaths by ensuring access to CPAP, effective antibiotics (by monitoring antimicrobial susceptibility regularly) and prompt and effective resuscitation.
4. To Strengthen the community based newborn care approach and community engagement.
5. To enhance the infection prevention and control activities.
6. To support all activities across the continuum of care.
7. To strengthen the political commitment for the neonatal mortality reduction to the level best.
8. Using the existing Health Extension platform, to strengthen the social mobilization activities.
